# Mesenchymal Stem Cells in the Treatment of Traumatic Brain Injury

**DOI:** 10.3389/fneur.2017.00028

**Published:** 2017-02-20

**Authors:** Anwarul Hasan, George Deeb, Rahaf Rahal, Khairallah Atwi, Stefania Mondello, Hany Elsayed Marei, Amr Gali, Eliana Sleiman

**Affiliations:** ^1^Department of Mechanical and Industrial Engineering, Qatar University, Doha, Qatar; ^2^Biomedical Engineering and Department of Mechanical Engineering, American University of Beirut, Beirut, Lebanon; ^3^Department of Biomedical and Dental Sciences and Morphofunctional Imaging, University of Messina, Messina, Italy; ^4^Biomedical Research Center, Qatar University, Doha, Qatar

**Keywords:** mesenchymal stem cells, central nervous system, traumatic brain injury, bone marrow, neurons

## Abstract

Traumatic brain injury (TBI) is characterized by a disruption in the normal function of the brain due to an injury following a trauma, which can potentially cause severe physical, cognitive, and emotional impairment. The primary insult to the brain initiates secondary injury cascades consisting of multiple complex biochemical responses of the brain that significantly influence the overall severity of the brain damage and clinical sequelae. The use of mesenchymal stem cells (MSCs) offers huge potential for application in the treatment of TBI. MSCs have immunosuppressive properties that reduce inflammation in injured tissue. As such, they could be used to modulate the secondary mechanisms of injury and halt the progression of the secondary insult in the brain after injury. Particularly, MSCs are capable of secreting growth factors that facilitate the regrowth of neurons in the brain. The relative abundance of harvest sources of MSCs also makes them particularly appealing. Recently, numerous studies have investigated the effects of infusion of MSCs into animal models of TBI. The results have shown significant improvement in the motor function of the damaged brain tissues. In this review, we summarize the recent advances in the application of MSCs in the treatment of TBI. The review starts with a brief introduction of the pathophysiology of TBI, followed by the biology of MSCs, and the application of MSCs in TBI treatment. The challenges associated with the application of MSCs in the treatment of TBI and strategies to address those challenges in the future have also been discussed.

## Introduction

Annually, around 10 million people worldwide suffer traumatic brain injuries (TBIs), which lead either to death or hospitalization (1). In the USA alone, an estimated 1.7 million people sustain a TBI each year, with death being the outcome for about 52,000 of those affected (2). Brain injuries may also have less pronounced effects that are harder to quantify and are classified as mild. Although deemed benign, even mild TBIs can lead to prolonged symptoms and long-term serious sequelae (3–5). Patients often complain of headaches for weeks after the injury, and the risk of depression remains higher for decades (6). TBI could also increase the likelihood of suffering from Alzheimer’s disease or dementia in old age (7–9). In USA, the direct and indirect costs of TBI are estimated at over US$60 billion per year (10), and life years lost due to death and disability outweigh medical and rehabilitation costs by a factor of four (11).

Traumatic brain injury occurs when the brain is damaged by an external force (12). This force could result from a direct impact of the head with an object (bump, collision, assault) or from an indirect impact (whiplash, sudden acceleration–deceleration of the head). In both cases, the brain is subjected to forces that damage its neural structure and alter its function. The trauma causes a primary injury/damage to the brain that is followed by a secondary injury. The second injury is manifested in cascades of multiple pathophysiological mechanisms, including cytotoxicity, gene activation, oxidative injury, cerebral edema, and inflammation. The body’s response to the initial injury has protective and reparative roles as well as negative effects that prevent the completion of the healing process in the affected area (13). In order to protect the intact neural tissue from the destructive immuno-response, a physical barrier termed the glial scar is formed around the injured area to isolate it and prevent the spread of the inflammation to neighboring neurons and the surrounding area (14). To address this issue and in attempt to restore functionality to the afflicted neurons, researchers have investigated various restorative approaches, one of which is regenerative medicine. A major new focus of regenerative medicine involves the use of stem cells. Stem cells are progenitor cells that are found in various niches of the body. There are several types of stem cells that have been used in the treatment of various bodily injuries, from muscular, skin, and bone to liver, bladder, and neural cells. Endogenous stem cells, exogenous stem cells, embryonic stem cells, induced pluripotent stem cells, adult stem cells, and mesenchymal stem cells (MSCs), to name a few. The scope of this review paper is solely the use of MSCs in the treatment of TBI. MSCs are multipotent stromal cells that can be extracted from all tissues (15) and have been shown to differentiate into various cell lines, not solely mesenchymal ones (16–18). Due to the relative ease with which these cells can be obtained, the abundance of their sources (19–23), and their wide differentiation potential, there is a growing interest in their use for regenerative purposes. Moreover, MSCs have been shown to selectively migrate to and settle in injured tissue (24–30). This “homing” capacity has been exploited to circumvent the difficulties associated with delivery of stem cells into delicate sites, such as in the brain or the heart. Furthermore, MSCs help in the regeneration of injured tissue through their multi-lineage differentiation potential. In addition, injecting MSCs into injured tissue has been shown to reduce the natural immune response (31–35) and to promote the tissue’s own regenerative process by releasing helpful growth factors (31, 36–38).

The use of MSCs is a promising strategy for the treatment of TBI. Considerable advances in MSC’s application in neuronal regeneration have been made in recent years, particularly in the methods of preparation of these cells for successful implantation and subsequent brain injury recovery. Most of the *in vivo* studies in this direction have been limited to animal studies (39–42). Translating these treatments to humans remains a challenge due to various reasons (43) including the need for well-established and reliable grafting techniques. In addition, the lack of knowledge of the specific mode of action of MSCs (the way they target tissues, the role of paracrine factors, among others) still limits the successful implementation in clinical practice (25). On another note, the immunogenic aspects of MSCs after transplantation (32) and their correlation with tumors (44) are also among challenges not to be neglected.

In the current review, we discuss the state–of-the-art and the recent advances in the application of MSCs in the treatment of TBI. The review gives a brief introduction of the pathophysiology of TBI, followed by the biology of MSCs, and the application of MSCs in TBI treatment. We then present the challenges associated with the application of MSCs in the treatment of TBI, and the strategies to address those challenges in the future have also been discussed.

## Pathophysiology of TBI

**Figure 1:**
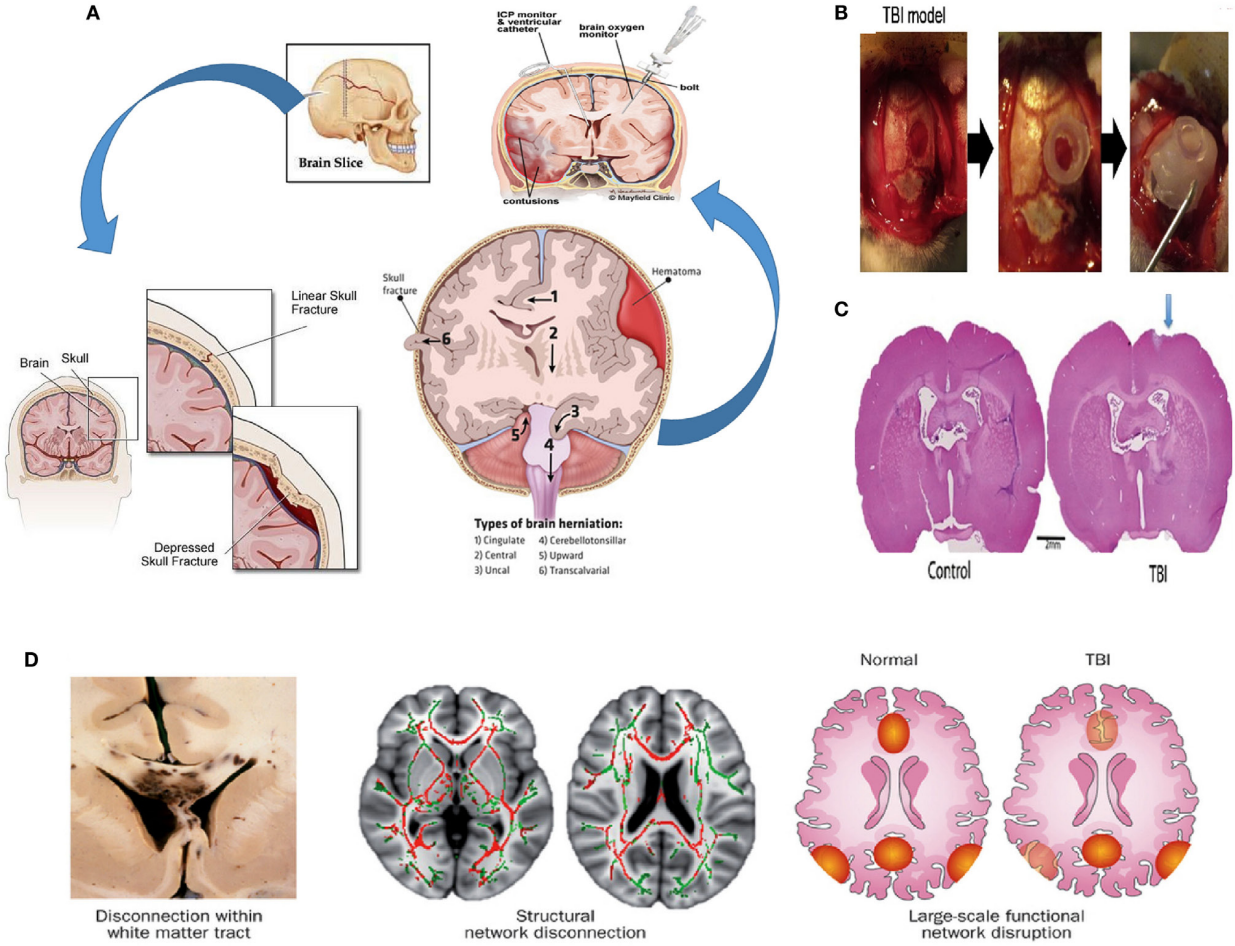
**Pathophysiology of TBI**. **(A)** Possible effects of TBI (hemorrhagic contusion, and midline shifts). A monitor can be inserted into the skull *via* surgical methods to reduce intracranial pressure (ICP) (45, 47–49). **(B)** Creating a TBI model in a rat; a 4.8 mm craniectomy was performed on the right parietal cortex (left panel), a plastic cylinder 4.8 mm in diameter was fixed at the craniectomy site (middle panel), a bone cement is placed to reclose the skull (right panel) (94). **(C)** A picture of the coronal rat whole brain section is shown for both the control case and the TBI case (50). **(D)** At the macroscopic scale, injuries can be noticeable in large white matter tracts, seen here in the leftmost bottom figure in a postmortem specimen with black regions of hemorrhage, indicating underlying damage. At the organ scale, damage to tracts interrupts long-distance communication between brain regions. The two pairs of axial human brain sections at the bottom center and bottom right illustrate the white matter microstructure with reduced fractional anisotropy due to a TBI (red structures in the bottom center) and intact structures (displayed in green). The damage could result in a disruption in the interaction between nodes of a brain network (illustrated as red and yellow regions in the bottom right figure) (51). Figures reproduced from Ref. (45, 47–49, 51, 52, 94) with permissions from Elsevier and Nature publishing groups and the International Journal of Critical Illness and Injury Science. Abbreviations: AMPA, α-amino-3-hydroxy-5-methyl-4-isoxazolepropionic acid; NMDA, *N*-methyl-d-aspartic acid; NO, nitric oxide; ROS, reactive oxygen species; TBI, traumatic brain injury.

The effects of TBI on the brain are numerous, and they can be divided between external and internal effects depending on the targeted area. Externally, the skull provides protection for the brain. Some cranial outcomes of TBI are scalp hematoma, hemorrhagic contusion, herniation, and midline shifts of the brain (Figure 1A) (45). Internally, the complex blood–brain barrier (BBB) structure separates the brain extracellular fluid from the circulating peripheral blood. The BBB maintains ion concentrations, regulates the flow of elements into the brain, and protects the brain from foreign elements (therapeutic and neurotoxic) circulating in the blood stream. In cases of brain injury, the BBB’s tight lock is compromised, allowing the passage of immune cells into the central nervous system (46).

Astrocytes that are part of the BBB are also particular key players in the brain’s defense response. After an injury, astrocytes enclose the damaged area to protect the rest of the brain creating the so-called *glial scar* (53). A consequence thereof however, is a much lower rate of inflow of macrophages (54), the immune cells responsible for removing the inhibitory myelin from the site, and, ultimately, the inhibition of the return to full functionality of the injured area (55).

While the incident of TBI is usually acute, general consensus is that there are two stages in the biological response to TBI. The first, or primary stage, is the physical damage to the neurons, the glial cells, the nerve fibers, and the BBB (56, 57). Damage to the neural structure involves linear and torsional forces. The linear force results from the direct acceleration–deceleration experienced during the collision. The linear force contributes to a torsional force among the neurons that leads to twisting and shearing injuries in the brain, significantly affecting the white matter fiber tracts that are especially vulnerable to injury. Axonal injury characterized by swelling and even complete severing of axons is a major and common result of TBI (51). The effect of TBI on neurons and the general neuronal tracts are shown in Figure 1D. Experiments by Johnson et al. showed that for *in vivo* and *in vitro* TBI models, myelinated fibers are more tolerant to mechanical strain than their unmyelinated counterparts (58). The susceptibility of nerves to injury is due to the viscoelastic nature of the nerve fibers. Although nerves do exhibit an elastic nature under gradual loading, their behavior is brittle under sudden sharp loads. Sharp sudden loads render the neuron brittle, resulting in the damage of axons.

Following the initial injury caused by the trauma, an intense local inflammation occurs, exacerbating the damage and expanding the site of injury to include neighboring neurons. The primary injury leads to ischemia, reducing the oxygen and glucose supply to the cells. This forces the cells to resort to anaerobic respiration and the accumulation of lactic acid. After the depletion of the ATP in the cells, the ion pumps in the cell membrane lose some functionality leading to the leakage of calcium ions into the cells and the mitochondria, leading to the formation of free radicals that cause more damage (52, 59–62). The inflammatory response consists of the recruitment and migration of leukocytes and microglia to the site of injury and the release of cytokines, some of which promote an inflammatory response (such as IL-6 and TNFα) and anti-inflammatory response (such as TGFβ and IL-10) in addition to oxygen radicals, nitric oxide, proteases, and other factors with cytotoxic effects, which, in turn, exacerbate neuronal death (52, 58, 60, 63). The complex cascade of resultant events is known as the secondary stage of TBI. In response to this cascade, astrocytes become hypertrophic and are activated building a physical barrier (the glial scar) isolating the site of injury and protecting the neurons that are still intact. The glial scar encloses an area containing inhibitory molecules that prevents the regrowth of neurons (14) and inhibits the repair of the BBB (64). Preventing the formation of the glial scar by inhibiting reactive gliosis might appeal as a treatment method (65). The astrocytes in the glial scar, however, encourage the survival of surrounding neurons by secreting various metabolites such as glucose, growth factors, and nutrients (14).

There exist many models of TBI. The lateral fluid percussion brain injury method process of modeling TBIs in rats is shown in Figures 1B,C (50, 66). Generally, they are separated into either penetrative injury or non-penetrative injury models. Penetrative injury models are implemented by the aforementioned lateral fluid percussion and controlled cortical impacts (CCIs). Non-penetrative injuries are caused by impact acceleration and weight drop models (67–71).

## Biology of MSCs

**Figure 2:**
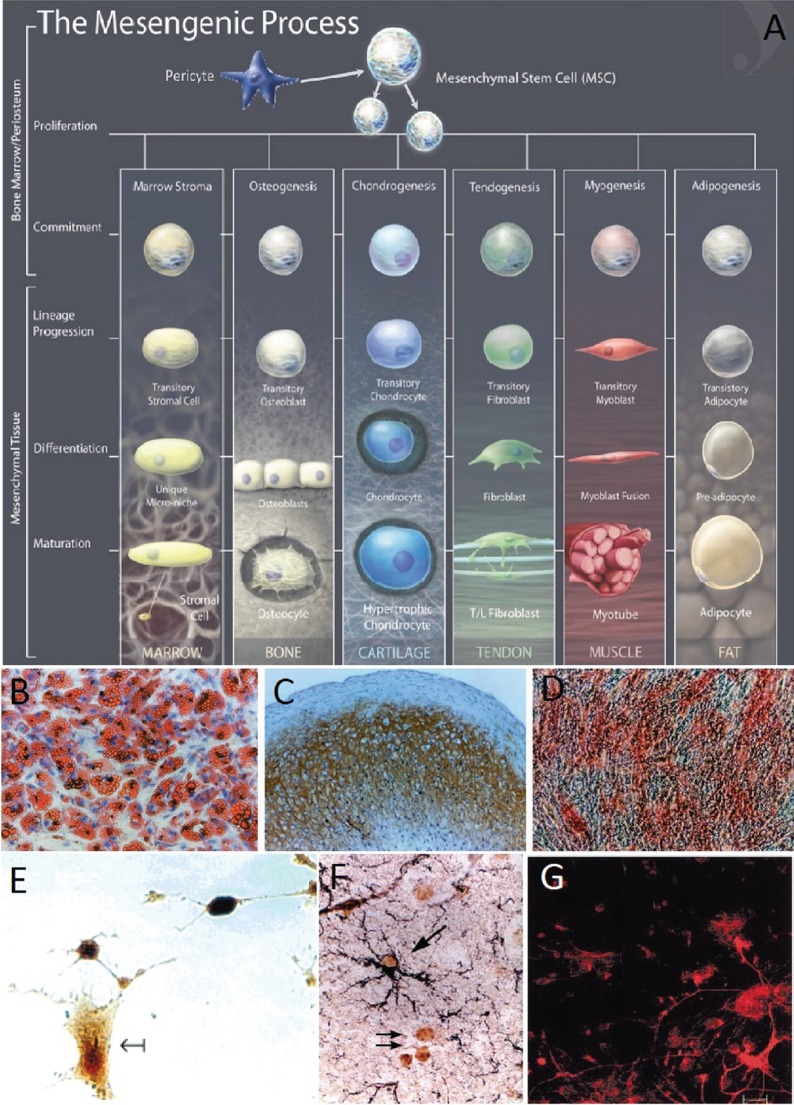
**(A)** The mesengenic process, typical lineages of mesenchymal stem cells (MSCs), and the stages of their differentiation (74). **(B–D)** Human MSCs (HMSCs) differentiate into the adipo, chondro, and osteocyte lineages (16). Adipogenesis is seen by the accumulation of natural lipid vacuoles that stain oil red **(B)**. Chondrogenesis is seen by staining with C4F6 monoclonal antibodies to type II collagen **(C)**. Osteogenesis is evident by the increase in alkaline phosphatase and calcium deposition typical of osteocytes **(D)**. **(E)** HMSCs differentiate in neurons and expressed high levels of the neuronal marker neuron-specific enolase (75). **(F)** Murine MSCs harvested and reinjected into neonatal murine brains differentiated into astrocytes. MSC-derived astrocytes in the hippocampus were labeled with anti-BrdUrd and anti-GFAP. The arrows indicate BrdUrd-labeled nuclei (76). **(G)** Murine BMSCs were labeled with fluorescent stain and cocultured with fetal midbrain cultures for 1 week. The red stain shows BMSC with astroglial cell marker GFAP (18). Figures reproduced from Ref. (16, 74–76) with permissions from the American Association for the Advancement of Science, John Wiley and sons, PNAS, and Elsevier publishing groups.

Mesenchymal stem cells are multipotent stromal cells that can be extracted from virtually any adult tissue (15) and have the potential to differentiate into a variety of cell types including the osteogenic, adipogenic, chondrogenic, and neural lineages (72–75) (Figure 2). It is required that MSCs be positive for CD105, CD73, and CD90 and negative for CD45, CD34, CD14, or CD11b, CD7α, CD19, and HLA-DR surface molecules (72). The expression or lack of expression of the aforementioned antigens ensures distinction between MSCs and other cells that would be present in an MSC culture. MSC’s therapeutic and restorative potential for TBI is evident from their ability to differentiate into neural cell lineages, to home to sites of injury, as well as to cross the BBB. In what follows, we will be discussing in more details each of these properties, which make MSCs particularly valuable for TBI treatment.

### Differentiation Potential of MSCs in Neural Cells

Azizi et al. showed that it is possible to engraft MSCs into the brain, where they survive and display migratory abilities similar to those of neural stem cells (NSCs) (77). Furthermore, whereas these MSCs could be stained with antibodies for collagen 1 before implantation, this characteristic was no longer maintained afterward. Their staining for fibronectin also decreased significantly 30 and 72 days later. The differentiation of MSCs into certain lineages and thus the expression of certain genes are explained by the potential of MSCs to acquire the phenotype of their host tissue (78).

Kopen et al. implanted immunodepleted MSCs into the lateral ventricle of neonatal mice (76). Consistent with previous observations, the distribution of the marked cells throughout the brain reflected a specific course of migration along defined routes. Furthermore, some of the cells migrated into the corpus striatum, the molecular level of the hippocampus, and the cerebellum, and differentiated into macroglia. This behavior of the cells was in line with the developmental stages of those parts of the brain. MSCs also migrated into parts of the brain undergoing neurogenesis, where they might have developed into new neurons. However, the cells did not migrate into regions of the brain where the population of neurons develops during embryogenesis. The implanted MSCs thus mimicked the behavior of neural progenitor cells in the postnatal murine brain.

Further evidence of the neural differentiation potential of MSCs was produced by Sanchez-Ramos and coworkers. They found that MSCs differentiated into neuron-like and glial-like cells both in the case of coculturing with primary neural cultures and without (18). Human MSCs (HMSCs) were also shown to differentiate, inside murine bone marrow, into the building blocks of the hematopoietic stem cell (HSC) niche, namely pericytes, myofibroblasts, reticular cells, osteocytes, osteoblasts, and endothelial cells (ECs) (79), the discussion on which is beyond the scope of the current review. The HSC niches maintained the progenitor cells in a quiescent state, protecting them from differentiation or apoptosis, and then controlled their proliferation and the release of their progeny into the vascular system (80, 81).

### Homing of MSCs at the Sites of Injuries

**Figure 3:**
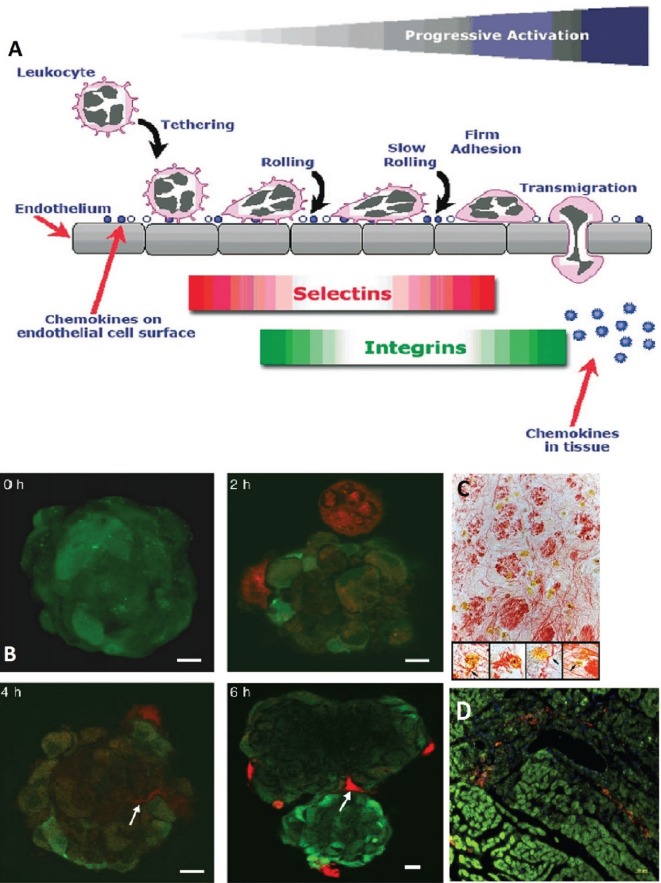
**(A)** A schematic showing how leukocytes transmigrate across the endothelium. Mesenchymal stem cells (MSCs) are likened to have similar patterns in their transmigration. The homing of leukocytes is affected by chemokines, selectins, and integrins released by the endothelial layer (24). **(B)** MSCs (red, stained with CellTracker Red) were cocultivated with endothelial spheroids (green, stained with CellTracker Green). The MSCs came in contact with the spheroid at 2 h and infiltrated it with plasmic podia (indicated with arrows at 4 and 6 h, scale bar 10 µm) (84). **(C)** MSCs in the reticular filament of the brain stem labeled with anti-BrdUrd, anti-GFAP, and anti-neurofilament (×400 magnification). The insets show neurofilament staining (re) in the cytoplasm of the BrdUrd stained (yellow) MSCs (×1,000 magnification) (76). **(D)** MSCs (DiI labeled, red) migrate to site of sub-endocardium myocardial ischemia. Damaged myocytes appear dark green and are more loosely organized than healthy myocytes (phalloidin labeled F-actin, green; and Hoechst labeled nuclei, blue) (29). Figures reproduced from Ref. (24, 29, 76, 84) with permissions from John Wiley and Sons, Elsevier, and PNAS publishing groups.

Mesenchymal stem cell homing is described as their ability to “arrest within the vasculature of a tissue followed by transmigration across the endothelium” (25). MSCs can migrate to sites of TBI injury (82, 83), and many mechanisms have been put forth to explain this behavior. For instance, López Ponte and colleagues showed that MSCs’ migration is influenced by several chemokines and growth factors (27). Another mechanism is the adhesion of MSCs to the endothelium of injured tissue due to the expression of vascular cell adhesion molecule (VCAM-1) (28–30). Figure 3 shows the homing mechanism of MSC to injury sites and infiltration and how they are similar to the homing mechanism of leukocytes. The figure shows how leukocytes have the ability to tether, roll, adhere, and transmigrate toward chemokines, selectins, and integrins secreted by the endothelial layer (24).

In *in vitro* coculturing, Rojas et al. reported a clear proliferation and migration of MSCs toward injured lung cell suspensions that did not occur when MSCs were cocultured with healthy lung cells (36). Barbash et al. found a considerably higher activity of infused MSCs in myocardial infarction (MI) versus sham-MI rats (26). They also compared the colonization sites of MSCs around injured myocardial tissue in rats. They identified the presence of donor cell at the infarcted border zone but none in intact myocardial tissue or in sham-MI rats. This suggests that MSCs can also locate injured cells inside otherwise healthy tissue.

As the amount of released markers could decrease over time, the efficiency of MSC migration could drop as time elapses after an injury. Barbash et al. registered a trend of increased presence of MSCs in rats in cases of MSC infusion after 2 days as compared to that after 14 days (26). However, the effectiveness of the infusion could depend on its location. The efficiency of the MSC homing capacity when infused into the left ventricular (LV) cavity versus that of intravenous infusion in rats with MI was compared. A considerably larger activity of MSCs was registered in the lungs after intravenous infusion compared to LV cavity infusion. Furthermore, the migration of MSCs to the site of injury in the heart was significantly more effective in the latter case.

### Immunosuppressive Properties of MSCs

In addition to their ability to differentiate into cells of various lineages and their tendency to home or migrate toward the sites of injuries, the immunosuppressive properties of MSCs have resulted in growing interest in their potential clinical applications. For example, the addition of MSCs from both autologous and allogenic sources to an *in vitro* mixed lymphocyte reaction led to a suppression of the proliferative response of the lymphocytes (34, 35). This effect was further amplified with increased numbers of MSCs. Furthermore, the suppressed lymphocytes were found to recover their properties when stimulated in the absence of MSCs (35). *In vivo*, the addition of MSCs to an allogenic skin graft also delayed the time of rejection from 7 to 11 days (34). These findings show that MSCs have immunosuppressive properties. This effect of MSCs could be exploited to help in reducing the effects of the secondary insult of TBIs.

### Regenerative-Aiding Effects of MSCs

Mesenchymal stem cells have been found to facilitate the injured tissue’s own regenerative process. MSCs implanted into mouse hippocampus were found to enhance the proliferation, migration, and differentiation of native NSCs (37). Chemokines released by MSCs might have themselves influenced the NSCs or done it indirectly through activating the surrounding astrocytes. A decreased expression of inflammation-associated cytokines was also reported in lung tissues treated with MSCs, which facilitated the natural repair of the injured tissue (36).

A common devastating outcome of TBI is the damage and undermining of the BBB. Menge et al. reported that MSCs upregulate the expression of the TIMP3 gene in TBI mice (43). The TIMP3 protein was shown to contribute to restoring the BBB to function through decreasing its permeability and reinforcing the junctions between ECs.

### Crossing of the BBB by MSCs

The ability to cross the BBB is important for neurotherapeutic drugs for their proper efficacy. Over the last decades, several strategies and technologies that enable access through the BBB have been investigated (85, 86). Recent research has indicated that MSCs might already possess the ability to cross the EC barrier of the BBB (87). Steingen et al. identified the mechanisms through which this occurs (84). After coming into the contact with the endothelium, MSCs exit the blood stream and integrate into the endothelium through the use of the adhesion molecules VCAM-1/VLA-4 and β1 integrin. After crossing the endothelial barrier, MSCs invade the host tissue through the use of plasmic podia (84). In the brain, Matsushita et al. reported that MSCs were able to cross the BBB through paracellular pathways (88), despite the presence of tight junctions that would normally inhibit such passage. Similar to lymphocytes, MSCs seem to influence tight junction barrier properties leading to their temporary abolishment. The MSC’s ability to cross the BBB is a primary cause of its appeal as a TBI treatment method.

## Reported Results in Decreasing TBI Sequelae

There is growing evidence supporting the efficiency of using MSCs in alleviating the severe consequences of TBI. In this regard, several studies have reported the potential mechanisms by which MSCs might enhance the function of patients’ nervous systems. In one study, it was found that MSCs differentiate into neuron- and astrocyte-like cells when transplanted into rats with TBI (39). This was demonstrated by the existence of the neuronal nuclear antigen and glial fibrillary acidic protein in the parietal lobes of the studied cells. Furthermore, researchers reported that this differentiation enhanced neural growth, promoting sensory and motor functions improvement (39, 89, 90). These results hold promise for the potential of MSCs in the treatment of TBI. Table 1 summarizes animal and clinical trial breakthroughs and findings concerning the therapeutic effect of MSCs in treatment of TBI and its sequelae.

**Table 1:** **Summary of studies of MSC biological properties and their therapeutic effects on TBI**.

Animal trials					
Mesenchymal stem cell (MSC) source	Traumatic brain injury (TBI) model	Animal	Administration	Result	Reference
Immune-depleted MSCs	N/A	Neonatal mice	Implanted into the lateral ventricle	Migration of MSCs into different part of the murine brain, some cells also underwent neurogenesis, where they developed into neuron	(76)
Human MSCs (HMSCs)	N/A	Mice	Injection into bone marrow	HMSCs differentiate into the building blocks of the hematopoietic stem cell niche, pericytes, myofibroblasts, reticular cells, osteocytes, osteoblasts, and endothelial cells	(79)
MSC	N/A	Rats	Infused in myocardial infarction (MI) and sham-MI	Infused MSCs demonstrated higher activity in MI being able to locate injured cells within healthy tissues. Homing was not observed in Sham-MI rats or healthy rats	(26)
MSC	N/A	Baboon	Addition of MSCs to an allogenic skin graft	MSCs displayed immunosuppressive properties by delaying rejection time from 7 to 11 days by suppressing the proliferative response of lymphocytes	(34)
MSC	N/A	Mice	Implantation into hippocampus	MSCs facilitate the regenerative process of the injured neural tissue by enhancing proliferation, migration, and differentiation of native neural stem cells	(37)
MSC	N/A	Rats	Transmigration in the brain	MSCs exhibited their ability to penetrate the blood–brain barrier (BBB) *via* paracellular pathways	(88)
Human UMSC	Controlled cortical impact (CCI)	Mice	Infusion into the cerebral ventricle	Mice exhibited improved motor skills after 35 days of TBI	(90)
HMSCs	CCI	Rats	Transplantation in the brain	MSCs decreased TBI sequelae by their inherent capability to differentiate into neuron- and astrocyte-like cells	(89)
HMSCs	FPI	Rats	Intravenous injection	MSCs mitigated TBI effects by reducing neuronal cell loss and apoptosis, and increasing the production of the vascular endothelial growth factor	(40)
Hypoxic-preconditioned MSCs	FPI	Rats	Rat brains were treated with hypoxic and normoxic-preconditioned MSCs	The MSCs increased their growth factor secretion due to hypoxia preconditioning	(91)
MSCs	CCI	Rats	Transplantation	MSCs with collagen scaffolds enhanced the survival of cells in rats with experimental TBI	(41)
MSCs	CCI	Rats	Topical application to the surface of the brain	MSCs with fibrin increased the adhesion efficiency of MSCs to the cortical brain surface and provided a scaffold for the increase of the MSCs before they penetrated the white matter to migrate to the site of TBI	(92)
BMSCs	Weight drop model	Rats	Transplantation with administration of exogenous basic fibroblast growth factor (bFGF)	Exogenous bFGF enhances the growth of transplanted cells, for the regeneration of tissue. And, rats following TBI exogenously supplied with bFGF recovered more quickly than rats without bFGF	(93)
MSCs combined with mannitol	FPI	Rats	Intra-arterial transplantation	Mannitol disrupts the BBB, which allows more MCSCs to be detected in injured brain tissues as compared to MSCs with glycerol or phosphate-buffered saline	(94)
UMSCs	Weight drop method	Rats	Increased ability to survive and migrate in rat cerebral tissues	Higher improvement in neurological function when rats received brain-derived neurotrophic factor gene-modified UMSCs due to an increase in the MSCs ability to survive and migrate in rat cerebral tissues	(95)
**Clinical trials**					
**MSC source**		**Age of patients (years)**	**Administration**	**Result**	**Reference**
Autologous MSCs		6–55	Transplanted cells at site of brain injury	MSCs enhanced neurological recovery by increasing the engraftment efficiency of transplanted cells	(96)
MSCs		6–10	Intravenous injection	7 of 10 patients showed improvement on Glasgow Coma Scale	(97)

Other studies have reported that the intravenous administration of secretome derived from HMSCs led to a decrease in the number of apoptotic neural cells promoting vascular endothelial growth factor (VEGF) release (40, 91, 98). These results support the idea that MSC-driven neural regeneration could restore neural function (99, 100). Factors secreted by MSCs include glial cell line-derived neurotrophic factor, brain-derived neurotrophic factor (BDNF), nerve growth factor (NGF), and VEGF. MSC cultures in supernatants from rat brains subjected to closed TBI also showed increased BDNF, NGF, and VEGF, in addition to an increase in hepatocyte growth factor (HGF). The large number of factors secreted can promote self-repair of residing tissue cells.

Furthermore, several preclinical trials investigating the use of MSCs in TBI models have shown the migration of cells away from the lesion site and subsequent survival of MSCs, as well as their differentiation into neurons and astrocytes, leading to enhanced motor function (101).

It is important to mention that it may be possible to administer factors produced by MSCs to improve the state of traumatically injured brains without transplanting the cells themselves. The potential recovery of neural function has been reported not to be due to MSCs replacing the dead neural cell, but rather to the fact that local progenitor cells are stimulated following the MSCs’ secretion of soluble factors, which in turn leads to the survival of the neural progenitor cells and subsequently their differentiation (31).

## Genetic Modification and Other Preconditioning of MSCs before Delivery to TBI

Recent research has highlighted the possibility of genetically modifying MSCs for the purpose of producing soluble growth factors, as well as cytokines and chemokines (98). These soluble factors are capable of enhancing the survival of stem and neuronal cells. For instance, neurotrophic factors secreted by MSCs have been found to promote angiogenesis and neurogenesis in the injured brain, thus enhancing the multiplication of neuronal cells at the damaged site (39).

**Figure 4:**
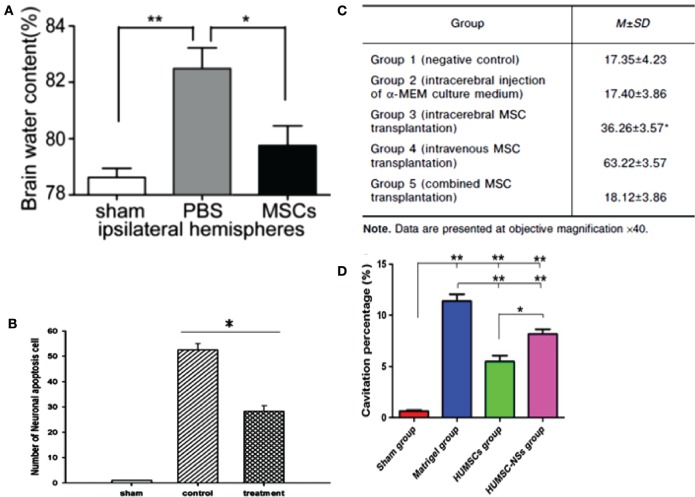
**(A)** Ipsilateral hemisphere brain water content of rats was analyzed 72 h after injury using a weight drop device. Rats treated with mesenchymal stem cells (MSCs) had significantly lower water content than rats injected with phosphate buffer solution (PBS) only (*n* = 6 per group, *p = 0.05, **p = 0.01) (99). **(B)** Rats impacted with traumatic brain injury (TBI) using fluid percussion injury and treated with MSC secretome showed a lower number of neuronal apoptosis cells compared to TBI rats with no treatment (*n* = 8 per group, *p < 0.05) (40). **(C)** Comparison of mean width of astroglial scar in TBI rats with different methods of MSC transplantation (100). **(D)** Cavitation percentages in different groups of rats subjected to TBI using a weight drop device. Rat groups were sham, matrigel treated, human UMSC, and human UMSC-derived neurospheres. Rats treated with human UMSC showed major improvement relative to the other groups (*p = 0.05, **p < 0.001) (102). Figures reproduced from Ref. (40, 99, 100, 102) with permissions from Wolters Kluwer Health and Springer publishing groups.

**Figure 5:**
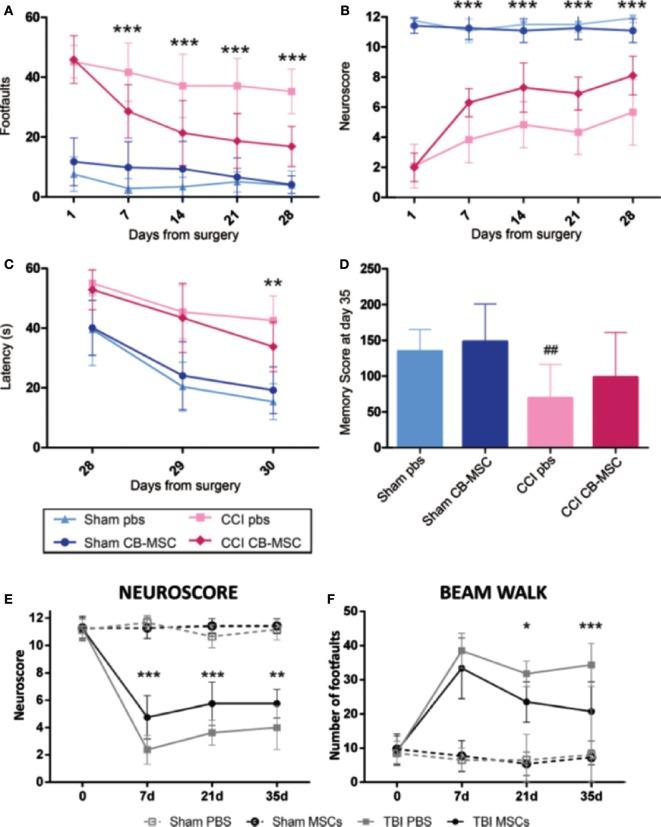
**The protective and recuperative effects of mesenchymal stem cell (MSC) on mice impacted with traumatic brain injury (TBI) *via* a controlled cortical impact (CCI) is observed compared to mice that suffered CCI but were only treated with phosphate buffer solution (PBS)**. **(A–D)** UMSC had protective effects on the sensorimotor and cognitive function of TBI mice. TBI mice that only received PBS showed motor function deficit assessed by beam walk [**(A)**, *n* = 6–18, ***p < 0.001] and Neuroscore [**(B)**, *n* = 16–18, ***p < 0.001]. Mice that received UMSC treatment showed attenuation of their motor deficits after the injury by 7 days and persisted until 28 days. UMSC-treated mice showed better learning capabilities assessed by the latency to locate the hidden platform in the Morris water maze 28–30 days after the surgery [**(C)**, *n* = 16–18, **p < 0.01] Significant improvement was seen in locating the hidden platform in UMSC-treated rats on day 3, indicating better learning capabilities [**(D)**, *n* = 16–18, ^##^*p* < 0.0] (90). **(E,F)** Infusion of MSCs in mice with CCI TBI-induced early and persistent improvement of the mice sensorimotor deficits as assessed by Neuroscore [**(E)**, *n* = 8, **p < 0.01, ***p < 0.001] and beam walk tests [**(F)**, *n* = 8, *p < 0.05, ***p < 0.001] (89). Figures reproduced from Ref. (89, 90) with permissions from Wolters Kluwer Health and Springer publishing groups.

Some recent studies have highlighted the therapeutic potential of the secretome of modified MSCs for the survival of neuronal cells (40, 91). The therapeutic efficiency of MSCs was improved by preconditioning these cells under hypoxia, in addition to using collagen delivery metrics and scaffolds, and encapsulated MSCs (EMSC). The intravenous injection of secretome from normoxia-preconditioned HMSCs can attenuate TBI by reducing neuronal cell loss and apoptosis in addition to increasing the production of VEGF. This was traced by immunofluorescence staining in TBI-induced rats (40). However, MSCs cultured in hypoxia were more effective than their normoxic counterparts in inducing the expression of both HGF and VEGF in cultured cells. It was also demonstrated that treating experimental TBI rats with hypoxia-preconditioned MSC secretome resulted in significant improvements in their motor functions as well as in their cognitive functions and neurogenesis. Furthermore, the rats treated with hypoxic-precondition MSCs showed signs of reduced brain damage compared to rats treated with the normoxic-preconditioned MSC secretome. Taken together, these findings suggest that the preconditioning of MSCs under hypoxia can enhance the therapeutic potential of the secretome, mainly due to increasing the secretion of growth factors from these cells (91). The recuperative effects of MSCs on TBI sequelae are shown in Figures 4 and 5.

The use of collagen delivery matrices has been reported to improve MSCs therapy by promoting the retention of human bone marrow MSCs (BMSCs) in the lesion site and limiting their distribution in the transplanted region. Collagen scaffolds also enhanced cell survival in rats with experimental TBI and improved brain metabolism, as detected by positron emission tomography, when compared with rats into which the stem cells were transplanted without collagen scaffolds (41).

The use of EMSCs rather than naked MSCs has been shown to reduce neuronal cell loss from the hippocampus and cortical neuronal and glial defects in CCI rat models (40). These therapeutic effects were further improved by designing EMSCs transfected to produce the glucagon-like peptide-1 (GLP-1), which was present in increased concentration in cerebrospinal fluid in rats treated with these GLP-1-secreting EMSCs (42).

The topical application of MSCs to the surface of the brain, as compared to systemic delivery of MSCs, can allow MSCs to migrate more efficiently and specifically to the TBI site, where they can replenish injured neurons and secrete various anti-inflammatory, immunomodulatory, and neurotrophic cytokines that facilitate neuronal regeneration. Interestingly, the topical application of green fluorescent protein (GFP)-expressing MSCs (GFP-MSCs) combined with a thin layer of fibrin was shown to increase the adhesion efficiency of the GFP-MSCs to the cortical brain surface (92). In addition, it provided a scaffold for the increase of the GFP-MSCs before penetrating the white matter to migrate to the cortical surface of the site of TBI.

Furthermore, the administration of exogenous basic fibroblast growth factor (bFGF), which enhances the proliferation of NSCs *in vitro* and *in vivo*, can promote BMSC transplantation-associated functional recovery in rats after TBI. Experimental data have provided evidence that exogenous bFGF enhances the growth of transplanted cells, which is necessary for the regeneration of neural tissue (103, 104). Moreover, rats exogenously supplied with bFGF following TBI recovered more quickly as compared to other groups of rats that did not receive bFGF (93).

Intra-arterial transplantation of MSCs combined with mannitol has been proven to be an effective treatment in experimental TBI models. Specifically, mannitol results in an increased disruption of BBB, which allows more MSCs to be detected in injured brain tissues as compared to MSCs with glycerol or phosphate-buffered saline (94).

In addition, umbilical cord MSCs (UCMSCs), which express a genetically modified BDNF, attenuate neurological deficits in rats with TBI because these cells have increased ability to survive and migrate in rat cerebral tissues. In fact, rat models of cerebral contusion in the motor-sensory cortex showed much higher improvement in neurological function when they received BDNF gene-modified UCMSCs than when they received UCMSCs alone (95, 102).

Notably, BMSCs labeled with superparamagnetic iron oxide *in vitro* can be tracked by susceptibility weighted imaging (SWI) sequence to study their survival and location in a rat model of TBI (105). SWI sequence might be a valuable tool in demonstrating the migration and distribution of the labeled BMSCs in the brain of TBI animals.

## MSC Therapy of TBI in Humans

Furthermore, a recent study has shown that oxidative stress production can be significantly manipulated by HMSCs, promoting cell migration and thus contributing to brain recovery following injury (106). Taken together, these studies demonstrate that the use of MSC-based approaches could serve as treatments for patients suffering from TBI. One study by Cox et al. implanted MSC into 10 children that had a TBI injury with a Glasgow Coma Scale (GCS) score between 5 and 8 and monitored them over the course of 6 months (97). In seven children, the outcome was positive showing improvement on the GCS. The other three children did not show a significant improvement to their quality of life. None of the children died or suffered adverse effects due to the use of the MSCs in their treatment. Subsequent to that study MSCs were studied to treat brain strokes in adults, presenting favorable results (107).

Tian et al. showed that the use of MSCs have a window of efficacy after the onset of TBI (108). MSCs were implanted *via* lumbar puncture into 97 patients, 24 in a permanent vegetative state. Different patients had different time span between the TBI injury and their treatment. The study showed that the efficacy of MSC treatment is increased the earlier it is implemented. Patients who underwent the therapy clos e to the date of their injury showed better signs of improvement.

The delivery of autologous mesenchymal stromal cells to patients with TBI has been shown to be a safe and practical procedure that can potentially enhance neurological recovery by increasing the engraftment efficiency of transplanted cells at the site of brain injury (96). The procedure consisted of administering 10^7^–10^9^ cells directly into the injured area of the brain during a cranial operation followed by the administration of 10^8^–10^10^ cells using intravenous infusion. This method renders this type of treatment feasible for facilities with ordinary equipment and procedures. The procedure was conducted on seven human patients (seven males and one female). None of the patients died or had any adverse effects due to the procedure conducted, although one patient experienced two episodes of epilepsy in the first 2 months. The Barthel index score of all patients increased as the 6 months of treatment progressed.

## Challenges

Despite the several lines of evidence that support the great potential of MSCs in the treatment of TBI, a number of obstacles to the success of this approach remain. For instance, one major drawback of the use of MSCs is the limited knowledge on the way these cells target specific tissues (25). Karp and Teo addressed the problem of targeting MSCs to the intended tissues (25). Importantly, they highlighted the gap of knowledge that exists concerning the relative importance of the effects caused by MSCs engrafted locally and of those engendered from paracrine factors that are secreted and that also diffuse to the target tissues. Paracrine factors have been shown to have a positive role in the healing capacity of MSCs (106). However, the precise process by which MSCs are capable of regenerating defective tissues still needs to be understood.

Another critical issue that hampers the successful use of MSCs for the treatment of TBI is their potential correlation with tumors. Djouad et al. reported that the injection of MSCs might lead to suppressing the patient’s antitumor response (109). To explain this mechanism, Bartholomew et al. suggested that MSCs have an important role in suppressing the lymphocyte proliferation *in vitro* (34) causing a deficiency in the immune response. While this might be a favorable effect in the case of skin graft survival, the use of MSCs in treating TBI would require extra precaution, particularly to avoid the probable rise of any tumor in long-term cultured MSCs (44). In addition, there is now clear evidence that MSCs exhibit immunosuppressive effects under inflammatory conditions (34, 35). Therefore, given that secondary mechanisms following TBI include a severe inflammatory response (110), the employment of MSCs in the treatment could give rise to unintended complications (32).

Kim et al. reported that while the transplanted stem cells in the animal models survived for a long period of time, not enough stem cells differentiated into neurons and astrocytes to be able to replace the tissue that is damaged. This motivated them to look into the use of HMSCs and track their effects on functional recovery (111). In this study, rats were intravenously treated with HMSCs 24 h after TBI. Neurological function was significantly recovered in the group that was treated with HMSCs 15 days post-TBI in comparison to the placebo group treated with saline. NGF, BDNF, and neurotrophin-3 levels showed an increase in expression after 2 days of treatment, though the expression decreased as time passed. The study shows that in the acute phase of TBI after injury, treatment with HMSCs can enhance the neurological functional outcome since the upregulation of the neurotrophic factors leads to a decrease in neural cell apoptosis.

As mentioned previously, Tian et al. showed that the use of MSCs has a window of efficacy after the onset of TBI. The study showed that the efficacy of MSC treatment is increased the earlier it is implemented. Patients who underwent the therapy close to the date of their injury showed better signs of improvement. An important challenge to overcome, therefore, is the effect of time on the efficacy of the treatment.

Another significant challenge in studying the effect of MSCs in the treatment of TBI in humans, is to have a defined protocol to gage the efficacy of the various studies against each other. The location of where the MSCs are harvested, the severity of the TBI, and the quantity of MSCs are just some parameters that are to be defined and implemented across all clinical studies.

## Conclusion and Future Prospects

The use of MSCs in treatment of TBI has gained enormous interest over the last decade. This is because, MSCs are relatively easy to harvest, they elicit no immune response, and they can differentiate into cells of neuronal lineages, thereby helping post-TBI repair of neural tissues. Their prospective role in healing TBI relies more on their effects on the cells in the host tissue. They aid in decreasing the inflammation in the host tissue, as well as encourage recuperation and the regeneration of severed nerves (112, 113). Given that MSCs also have a tendency of homing near injury sites and an ability to migrate across the EC layers of injured tissue, more importantly the BBB, their use would circumvent one of the major hurdles in treating TBI, which is selective and targeted delivery to the injured tissues.

These advantages, however, are yet to be exploited to full effect. Further research is still required for better understanding the pathophysiology of TBI, the mode of action of MSCs and their trophic effects on inflamed host tissue, and the mechanisms of functions of MSCs in TBI *in vivo*. Furthermore, better understanding of the mechanisms of MSC homing in TBI affected regions is also important in order to be able to employ them efficiently in clinical applications. Further research is needed to differentiate between the respective roles of paracrine effects (growth factor, for example), transdifferentiated or progenitor cells, and many other factors in tissue repair (38). Studies could be conducted to study if MSC secretomes are solely required for the regenerative effects of MSC therapy, or MSCs are essential to the success of therapy. Recently, there have been concerns over probable role of MSCs in the development of cancer and autoimmune diseases. This possible side effect requires further investigation (109). On another note, the *in vivo* studies reported so far on application of MSCs in TBI have mostly been in the order of days or months (94, 114). Longer term *in vivo* studies are required before a widespread clinical application of MSCs in TBI.

## Author Contributions

AH initiated the review, planned and prepared the outline, recruited other authors, and contributed both in writing and editing the manuscript as well as taking the overall lead. All other authors contributed to this paper by writing some sections and reviewing the complete draft.

## Conflict of Interest Statement

The authors declare that the research was conducted in the absence of any commercial or financial relationships that could be construed as a potential conflict of interest.
